# Oxytocin, a Novel Treatment for Methamphetamine Use Disorder

**DOI:** 10.3390/neurolint14010015

**Published:** 2022-01-30

**Authors:** Amber N. Edinoff, Elliot Thompson, Chandler E. Merriman, Mark R. Alvarez, E. Saunders Alpaugh, Elyse M. Cornett, Kevin S. Murnane, Rachel L. Kozinn, Mila Shah-Bruce, Adam M. Kaye, Alan D. Kaye

**Affiliations:** 1 Department of Psychiatry and Behavioral Medicine, Louisiana State University Health Shreveport, Shreveport, LA 71103, USA; elliot.thompson@lsuhs.edu (E.T.); kevin.murnane@lsuhs.edu (K.S.M.); 2 School of Medicine, Louisiana State University Health Shreveport, Shreveport, LA 71103, USA; cem002@lsuhs.edu (C.E.M.); mra001@lsuhs.edu (M.R.A.); 3 Department of Anesthesiology, Louisiana State University New Orleans, New Orleans, LA 70112, USA; ealpau@lsuhsc.edu; 4 Department of Anesthesiology, Louisiana State University Shreveport, Shreveport, LA 71103, USA; elyse.bradley@lsuhs.edu (E.M.C.); alan.kaye@lsuhs.edu (A.D.K.); 5 Louisiana Addiction Research Center, Shreveport, LA 71103, USA; 6 Department of Pharmacology, Toxicology & Neuroscience, Louisiana State University Health Shreveport, Shreveport, LA 71103, USA; 7 Department of Anesthesiology and Pain Management, University of Texas Southwestern, Dallas, TX 75390, USA; rkozinnmd@gmail.com; 8 Department of Obstetrics and Gynecology, Louisiana State University Shreveport, Shreveport, LA 71103, USA; mila.shahbruce@lsuhs.edu; 9 Department of Pharmacy Practice, Thomas J. Long School of Pharmacy and Health Sciences, University of the Pacific, Stockton, CA 95211, USA; akaye@pacific.edu

**Keywords:** methamphetamine, oxytocin, substance abuse, treatment

## Abstract

The treatment of substance abuse with oxytocin is a novel approach to a challenging public health issue that continues to contribute to a growing economic cost for societies worldwide. Methamphetamine addiction is one of the leading causes of mortality worldwide, and despite advances in understanding the neurobiology of methamphetamine addiction, treatment options are limited. There are no medications that the Food and Drug Administration currently approves for stimulant use disorder. Off-label use of therapies for stimulant misuse include antidepressants, anxiolytics, and milder stimulants as replacement agents. Due to the shortcomings of these attempts to treat a complicated psychiatric disorder, recent attention to oxytocin therapy (OT) has gained momentum in clinical studies as a possible therapy in the context of social stress, social anxiety, social cognition, and psychosis. Oxytocin produces enhanced connectivity between cortical regions. The results from studies in rodents with OT suggest that central neuromodulation of oxytocin may be beneficial across transition states of stimulant dependence and may alleviate intense withdrawal symptoms. Studies of oxytocin in the context of other drugs of abuse, including cocaine, cannabis, and alcohol, also support the potential of oxytocin to treat stimulant use disorder, methamphetamine type. Methamphetamine abuse continues to be a significant cause of distress and dysfunction throughout the world. The effects of oxytocin on methamphetamine use outlined in this review should act as a catalyst for further investigation into the efficacy of treating stimulant use disorder, methamphetamine type with oxytocin in humans. More human-based research should initiate studies involving the long-term efficacy, side effects, and patient selection.

## 1. Introduction

The treatment of substance misuse with oxytocin is a novel approach to a challenging public health issue that continues to contribute to a growing economic cost for societies worldwide. Methamphetamine is a common drug of abuse and contributes to many detrimental health outcomes [1]. Drug addiction is one of the leading causes of mortality worldwide, and despite advances in understanding the neurobiology of drug addiction, treatment options are severely limited [2]. Current therapies for stimulant use disorder include antidepressant, anxiolytic, and mild stimulatory pharmacologic agents, though none are FDA approved. Additionally, alternative therapies such as behavioral and herbal remedies have led to limited therapeutic results and have been widely ineffective in clinical use [3].

Although there are currently many different treatments for substance abuse and, more specifically, methamphetamine use disorder, which remains to be a challenging disease to treat. For example, many cocaine rehabilitation programs have dropout rates of up to 50% due to the lack of effective pharmacotherapies that adequately treat withdrawal symptoms [3]. Due to the shortcomings of these attempts to treat a complicated psychiatric disorder, recent attention to oxytocin therapy (OT) has gained momentum in clinical studies as a possible therapy in the context of social stress, social anxiety, social cognition, and psychosis [4,5].

Endogenous oxytocin is a nine amino acid peptide that is produced by the hypothalamus and enters the peripheral circulation [6]. Peripherally, oxytocin promotes uterine contraction and lactation, during the intrapartum and postpartum period but additional receptors are located in the kidneys, pancreas, and heart. Furthermore, central oxytocin receptors have been located in the mesocorticolimbic system and other nuclei in the brain, including the medial and central amygdala (CeA), substantia nigra (SN), paraventricular thalamic nucleus, olfactory nucleus, hippocampus, brainstem, and the spinal cord, among others [6,7]. Oxytocin can also produce enhanced connectivity between cortical regions [8]. Modifying central neurotransmitter function in these regions is understood to attenuate neurobehavioral manifestations, including bonding, maternal, and stress-reducing behaviors, and contributes to the sensation of reward, stress, social affiliation, learning, and processing memories.

Due to recent developments in the understanding of oxytocin neurophysiology, targeting the central neuromodulation of oxytocin may induce improved symptomatic effects in treating methamphetamine use disorder. Due to the current advancements in oxytocin neurophysiology and modulation of behavior in the setting of chronic drug abuse, the medical community has brought further attention to oxytocin as a possible pharmacotherapy, which warrants further clinical investigations [7]. This review aims to look at the evidence regarding the use of oxytocin for the treatment of stimulant use disorder, methamphetamine type.

## 2. Methamphetamine

### 2.1. Epidemiology

The use of methamphetamine, a highly addictive psychostimulant, continues to grow with devastating effects throughout the United States and worldwide. The use of the drug soared in the 1990s, mainly in the midwestern and western parts of the USA, eventually reaching epidemic status in the early 2000s [9]. In an attempt to temper the use and production of methamphetamine, many state governments began limiting over-the-counter access to methamphetamine precursor products such as pseudoephedrine in 2004 [10]. The federal government followed closely behind with federal legislation to impose the same limits in 2006 [10]. These regulations did cause methamphetamine use and laboratory incidents to trend downward for a few years; unfortunately, the 12-month prevalence of methamphetamine use by persons 12 years and older increased by 195% from 2010 to 2018 in the USA [11]. The prevalence of methamphetamine use, in 2019, was 0.7% which showed no significant increase from 2018. Recent data from the National Survey on Drug Use and Health (NSDUH) regarding methamphetamine use in the USA concluded that the number of people with stimulant use disorder methamphetamine type was 1,048,000 in 2019 [12]. This is a significant increase as compared with the data from 2016, which showed 684,000 people with stimulant use disorder methamphetamine type [12]. Two million people 12 years or older reported using the drug in the past year [12]. Overall, methamphetamine has a strong presence throughout the United States, which indicates that this psychostimulant will continue to be a significant component of the drug epidemic for years to come.

### 2.2. Pathophysiology

Methamphetamine works through various mechanisms to increase the availability of catecholamine neurotransmitters, including dopamine and norepinephrine, in nerve terminals in the central nervous system (CNS) and peripheral nervous system (PNS) [13]. Amphetamines, including methamphetamine, act as substrates for the plasmalemma dopamine transporter (DAT), allowing admittance into dopaminergic pre-synaptic terminals [14,15]. After amphetamines are in the presynaptic terminal, they interact with vesicular monoamine transporter 2 (VMAT2) to trigger dopamine release into the cytosol, disrupting the pH balance [16]. Following the release, cytosolic dopamine concentrations remain high because amphetamines also interfere with the vesicular reuptake of the catecholamine via VMAT2 [17]. Catecholamines are metabolized by the mitochondrial enzyme monoamine oxidase (MAO); methamphetamine interferes with MAO function to inhibit the metabolism of catecholamines [18]. The result of increased availability and decreased metabolism is a massive abundance of catecholamines released in synaptic clefts, which can produce a variety of physiologic consequences including addiction, psychiatric changes, neurologic damage, cardiovascular damage, and gastrointestinal damage [19]. 

Multiple studies have linked long-term methamphetamine use to alterations of brain structure and biochemistry [20]. The common consequences of these changes have included impairments of memory, learning, language skills, motor skills, and visuo-constructional abilities; high rates of psychiatric manifestations including psychosis, anxiety, and depression have also been observed [21,22,23]. Methamphetamine also has been shown to deplete dopamine, serotonin, and their metabolites in several brain regions [24]. The possible mechanisms of methamphetamine-induced brain changes are neurotoxicity and inflammation [25]. Excess dopamine auto-oxidizes in the cytoplasm of neurons, enabling significant production of reactive oxygen species [26]. Subsequent oxidative stress leads to significant dysfunction of neurons, terminal degeneration, and apoptosis [27]. Psychostimulants also cause excitotoxic damage by stimulating glutamate release [26,28].

Methamphetamine is proposed to upregulate inflammatory processes, in part, by activating toll-like receptor 4 (TLR4) [29]. The TLR4 signaling pathway is a well-studied component of the innate immune system’s proinflammatory cytokines and chemokines [30]. Methamphetamine-induced TLR4 signaling increases NF-κB activation of microglia and mRNA expression for the proinflammatory cytokine IL-6 in the ventral tegmental area (VTA) [29]. The downstream effect of this TLR4-IL-6 signaling in the VTA is neuroinflammation and an elevation of dopamine in the NAc shell [29]. These mechanisms of dopamine-induced neurotoxicity and inflammation cause disruption of endogenous dopamine signaling by destroying post-synaptic neurons and dopamine terminals [26]. 

Long-term methamphetamine abusers have been found to have reduced striatal dopamine levels [31]. A further study concluded that the dopamine levels in the putamen were as severely diminished as liken to people with Parkinson’s disease [32]. Morphological changes visible on MRI also provide evidence for neurotoxicity from methamphetamine use. Prominent changes include decreased hippocampal sizes, enlarged striatum, and loss of grey matter in cingulate and limbic cortices [33,34]. 

#### 2.2.1. Current Treatment of Methamphetamine Use Disorder

Currently, there are many studies on the behavioral effects of a large variety of pharmacologic classes that target different regions of the brain. However, most of these studies have been limited to small clinical trials, and there are currently non-FDA-approved medications to treat cocaine or methamphetamine addiction [4,35]. Additionally, there has been no reliable evidence of any current pharmacologic agents that reduce psychological distress or physical symptoms associated with methamphetamine use [36]. Throughout these studies, numerous classes of medications target different receptors, including GABAergic and dopaminergic. Among others, they are central to the addiction process as its activity has been associated with reward processing in the brain [3]. Some of the psychotropic pharmaceutical classes studied include tricyclic antidepressants (TCAs), monoamine oxidase inhibitors (MAOIs), selective serotonin reuptake inhibitors (SSRIs), mood stabilizers, and dopamine agonists [4,35].

Additionally, many inconsistent effects have been found in treatment with propranolol as an anxiolytic, and bupropion and mirtazapine for associated depression. At the same time, most consistently, the benefits include mild stimulatory agonists including dexamphetamine and methylphenidate for amphetamine withdrawal and naltrexone and topiramate [35]. Furthermore, GABA enhancers, including topiramate, and more recently, N-acetylcysteine, have shown some marginal benefit and may be beneficial in reducing cravings and relapse prevention [35,37]. While many attempts to treat methamphetamine use pharmacologically have not been consistently significant, observing the behavioral effects due to central neuromodulation of these therapeutics has led to further advancements such as the understanding of the neurophysiology of substance abuse and central reward systems. The limitations of these treatments are the lack of clinical studies to evaluate their effectiveness in the general population.

The medical community is focusing on OT as an emerging treatment for methamphetamine use given the lack of consistent response of previous alternative therapies. Recent studies in rodents and humans suggest that OT may modulate substance-induced behaviors in the context of methamphetamine and cocaine use and even enhance prosocial behavioral responses to 3,4-methylenedioxymethamphetamine (MDMA, Ecstasy) [38]. Additionally, there are decreased measured levels of OT peptides in states of chronic withdrawal, and oxytocin receptors are upregulated. For this reason, central neuromodulation of oxytocin pathways through activation of a variety of glutamate and dopamine receptors, among other mechanisms, may reliably alleviate specific symptoms of methamphetamine withdrawal [7]. Since there is growing evidence of oxytocin modulation of behavior in social stress, social anxiety, social cognition, and psychosis, OT may provide benefit through similar mechanisms in the treatment of methamphetamine use [5]. 

#### 2.2.2. Oxytocin

The neuropeptide oxytocin is primarily known for its functions regarding parturition, lactation, and interpersonal attachment. Extensive evidence now supports oxytocin’s role in the complex physiology of addiction and the attenuation of anxiety, inflammatory, and stress responses [39,40]. Oxytocin is synthesized in the paraventricular nucleus (PVN) and supraoptic nucleus (SON) of the hypothalamus. Most of the synthesized peptide is transported to the posterior pituitary for storage and secretion. A portion of oxytocin is released centrally from the PVN and SON to act on receptors throughout the brain, including portions of the cortex, olfactory system, basal ganglia, limbic system, and brainstem [39,41,42]. Oxytocin has been linked to various physiological processes related to drug addiction, including the hypothalamic-pituitary-adrenal axis stress response, the central amygdala relating to anxiety and fear, and antinociception, autonomic regulation via the brainstem [43,44]. The abundance of oxytocin receptors throughout the CNS supports the growing information regarding the function of this neuropeptide. 

Research involving the intricate cycle of addiction has secured a place for oxytocin as a potential treatment for the disease. The addiction cycle includes drug consumption, intoxication, and reward; oxytocin interferes with these three stages via multiple proposed mechanisms. The promising effects of oxytocin on the drug addiction cycle can be partially attributed to its action on dopaminergic reward pathways, specifically the connection between the VTA and the NAc [45,46]. The hypothalamic PVN houses oxytocinergic projections that terminate in the NAc and other addiction-associated areas of the brain [47]. Administration of oxytocin reduces both the consumption of substances of abuse (SOA) and reward consequences while also decreasing some of the intoxicating effects of SOA in rodent studies [45,48,49]. The accumulating evidence that exhibits oxytocin’s interactions with neural substrates related to addiction makes this neuropeptide a promising treatment option for these destructive diseases. 

### 2.3. Mechanism of Action

Improved techniques in neurobiology have identified a variety of locations for oxytocin receptors and different projections in the brain. Many central oxytocin receptors are involved in mood and social behavior located in the mesocorticolimbic system’s reward processing region. These projections operate mostly through GABA neurotransmitter pathways. Additional effects on behavior, sensation, and perception may be mediated through centrally released oxytocin that projects to other, diverse brain regions. The regions include the medial amygdala and CeA, SN, paraventricular thalamic nucleus, olfactory nucleus, hippocampus, brainstem, and spinal cord. Other regions include the lateral mammillary nucleus, ventral pallidum, globus pallidus, basal nucleus of Meynert medial preoptic area, dorsal raphe nucleus, tubercle, and lateral septum may also be involved [6,7]. Although the diverse effects of OT neuromodulation in methamphetamine abuse are not well understood, recent findings are suggestive that oxytocin interacts with dopamine, glutamate, GABA, and vasopressin receptors in these central nuclei [6]. Even more so, oxytocin has also been shown to enhance connectivity between frontal and other cortical regions [8]. 

In terms of its role in substance abuse, the mechanism of oxytocin neuromodulation is thought to be through glutamate receptors, which remain active in the various regions involved in forming memories, learning, and reward processing [6]. Further findings suggest oxytocin neuromodulation resists alterations to glutamate–dopamine and glutamate–GABA-A interactions through drug-induced effects on glutamate transmission. Current theories suggest that these interactions enhance GABA’s inhibitory effects on glutamate and dopamine neurons and reduce mesocorticolimbic dopamine and corticolimbic glutamatergic pathways [6,7]. In addition to modification at the synaptic junction, OT has demonstrated physiologic effects on astrocyte function by reducing glial fibrillary acidic protein (GFAP) expression. GFAP is a protein that is increased in drug-induced states, can alter neural plasticity, and has associated neurotoxic properties. Reduced levels of GFAP ultimately result in reduced glutamate transmission through decreased GLT-1 transport of glutamate to the cell surface. Therefore, these findings suggest an additional explanation for oxytocin’s mechanism in attenuating drug-associated behaviors. Through restoring GFAP expression and, therefore, reversal of astrocyte function, OT may indirectly affect glutamatergic transmission opposing drug-induced behaviors [6].

## 3. Pre-Clinical Studies

Nearly all of the research into the behavioral and physiological effects of oxytocin on methamphetamine abuse has been performed on rat models, the results of which generally suggest that oxytocin attenuates the methamphetamine reward and reduces methamphetamine-induced hyperactivity [50,51,52,53,54,55,56]. Furthermore, these results were supported by Stauffer et al., the only human trial, to date, that investigated oxytocin’s effects on subjects with stimulant use disorder methamphetamine type [57]. Further discussion of notable research can be found below.

### 3.1. Subiah et al.

Subiah et al. devised a study to evaluate the effects of vasopressin and oxytocin on the mesolimbic system of rats following exposure to methamphetamine [50]. Investigators exploited the mesolimbic system’s connection to classical conditioning by evaluating whether vasopressin’s long-term facilitatory effect or oxytocin’s inhibitory effects could interfere with place preference induced by methamphetamine exposure [50]. 

The first part of the study was the initial acquisition period. The study subjects were divided into two groups: the treatment group was exposed to a chamber with saline and a separate room with a methamphetamine solution over several days for equal amounts of time, while the control group was conditioned to saline in both chambers for the entire acquisition period [50]. There were three phases of this period. In phase 1, each test animal was allowed access to the entire apparatus for 15 min. The amount of time was monitored, and an average time was used to determine the naturally preferred chamber for the animal [50]. During phase 2, on Days 1,3,5, and 7, rats in the methamphetamine group were given a dose of methamphetamine (2.5 mg/kg i.p.) and placed into the less preferred chamber for 50 min. This was to determine if place preference was dose dependent. Saline was injected on those days in the saline groups. On Days 2, 4, 6, and 8, rats received an equivalent volume of 0.9% saline before being confined to the preferred compartment for 50 min [50]. This was to ensure that the association with the non-preferred chamber was driven by methamphetamine. In phase 3 which was on Day 9, the partition separating the two chambers was removed and the subjects were allowed to freely access the entire apparatus for 15 min. The amount of time spent in each chamber was recorded [50]. 

The extinction phase is where the place preference is unpaired from the conditioned stimulus (i.e., administration of methamphetamine). The subjects were evenly distributed into groups sequentially exposed to saline and either oxytocin or vasopressin after this initial place preference test. After conditioning and the initial preferential placement, from Days 10 to 17, saline was alternately paired four times with each of the chambers once per day over eight days. In the oxytocin and vasopressin groups, oxytocin or vasopressin was administered instead of saline during this extinction phase. On Day 18, a second-place preference test was performed [50]. One group served as the control and was exposed only to saline throughout the extinction phase [50]. A day after the second-place preference test, three of the subjects were primed with a methamphetamine injection before all subjects underwent a third-place preference test (Day 19) [50]. Then, all subjects were decapitated, with their striata and hippocampi analyzed for dopamine and phosphorylated cyclic AMP response element-binding protein (pCREB); dopamine levels as an indicator for methamphetamine exposure and pCREB as an indirect indicator of learning and memory processes connected to addictive behaviors [50].

Whereas rats exposed to vasopressin in the extinction phase demonstrated no place preference changes with re-exposure to methamphetamine, those given oxytocin showed successful extinction and a statistically significant place preference during the reinstatement phase [50]. There was no significant difference between the oxytocin and vasopressin groups in terms of striatal dopamine level. Notably, there were significantly lower striatal dopamine levels between the oxytocin group and the methamphetamine-oxytocin group [50]. No significant difference in pCREB levels were noted across all study groups [50]. 

In all, these results suggest that methamphetamine induces a place preference, that vasopressin therapy following methamphetamine exposure plays a role in eliminating seeking behaviors, and that oxytocin may increase seeking behaviors with more potential for relapse [50]. This discrepancy was thought to be because oxytocin was unable to overcome the reinforcing effects on the brain when methamphetamine was reintroduced into the rat’s system. This harkens to the possibility that abstinence from methamphetamine may be needed for efficacy.

### 3.2. Qi et al.

Qi et al. investigated the effects of OT on conditioned place preference induced by methamphetamine in mice, and also evaluated the potential connection between glutamatergic neurotransmission in the reestablishment of methamphetamine-induced conditioned place preference [57]. 

After eliminating the mice that showed a habitual preference to one of two neutral chambers, the mice underwent conditioning, wherein the subjects underwent sequential exposures as follows: On even days, subjects were administer methamphetamine with confinement in the drug chamber of the conditioned place preference apparatus; on odd days, subjects were given saline and were restricted to the neutral chamber. All subjects were conditioned for the same amount of time in both chambers, which represented the conditioning phase of the study [57]. Following conditioning, the mice were subjected to another conditioned place preference trial following administration of either oxytocin or aCSF before being placed in the conditioned place preference apparatus [57]. Then, the study subjects had an intracerebroventricular cannula placed, and were injected with either oxytocin or artificial cerebral spinal fluid (aCSF) for five consecutive days, representing the extinction phase [57]. The subjects were, then, administered either oxytocin or aCSF before being primed with methamphetamine and re-evaluated for CPP [57]. Throughout the extinction and reinstatement phases, microdialysis of the mice brains was performed to detect the levels of glutamate in their medial prefrontal cortices (mPFC) [57].

Unsurprisingly, methamphetamine exposure resulted in a strong conditioned place preference not observed in the saline groups [57]. During the acquisition phase, higher dose oxytocin significantly reduced the subjects’ time spent in the methamphetamine paired chamber relative to the group dosed without oxytocin or with lower doses [57]. However, during the conditioned place preference following the conditioning phase, oxytocin demonstrated no significant effect on conditioned place preference expression following methamphetamine exposure, suggesting oxytocin played no role in the prevention of learned drug-seeking behavior [57]. The results showed that the oxytocin group demonstrated a faster time to extinction than the methamphetamine group, though there was no significant dose-dependent relationship [57]. Additionally, oxytocin demonstrated abolition of conditioned place preference reinstatement of methamphetamine, even following stressor induction [57]. 

In all, this study suggests that oxytocin partially attenuates the reward system activated by methamphetamine exposure—an inhibitory role that was demonstrably related to the glutamate neurotransmission [57]. Glutamate was increased in the mPFC in mice who received oxytocin.

### 3.3. Carson and Cornish et al.

Carson et al. devised an experiment to analyze the effect of dosed oxytocin on methamphetamine self-administration and relapse in rat models [49]. After allowing the study subjects to self-administer a methamphetamine solution until their response had stabilized, the treatment arm was treated with oxytocin of increasing doses before being allowed back into the experimental apparatus containing two levers, one giving methamphetamine. Following five days of either oxytocin or saline administration, the rats were left in their cages for ten days to allow for the extinction of the conditioned response [49]. Once the rats lacked observable methamphetamine-seeking behavior, the treatment and vehicle arms were injected intraperitoneally with methamphetamine before being placed back in the study apparatus This process was repeated following an additional extinction period but in which the treatment arm was administered oxytocin before the intraperitoneal methamphetamine [49].

Following this initial experiment, an additional experiment was devised to analyze the effects of oxytocin on methamphetamine-induced hyperactivity on drug-naïve rat models to account for potential confounds resulting from the diminished operant conditioning observed in the treatment arm [49]. After allowing for habituation to the new environment following the change of the study apparatus, the rats were divided into treatment, vehicle, and control arms, with their overall activity in the study apparatus monitored over regular intervals following administration of methamphetamine and pretreatment with either oxytocin, saline, or nothing [49].

The first experiment demonstrated a reduction in self-administered methamphetamine when those subjects were pretreated with either 0.3 or 1 mg/kg oxytocin, with a reduction in overall hyperactivity in the animals pretreatment with either 0.1, 0.3, or 1 mg/kg of oxytocin [49]. There was also no demonstrable difference in extinction rates among the treatment, vehicle, and control arms [49]. In all, these findings suggest there may be a role for oxytocin in the treatment of stimulant use disorder methamphetamine type in humans, though further study is warranted [49].

### 3.4. Carson and Hunt et al.

Taking their prior research one step further, with the effects of oxytocin on methamphetamine self-administration, conditioned place preference, and hyperactivity being well-established in prior rodent models, Carson et al. sought to identify specifically where in the brain oxytocin was producing its effects [51]. 

Rats were divided into treatment and control groups using a similar model as their prior research into oxytocin’s effects on reducing methamphetamine-induced hyperactivity. The treatment group was administered oxytocin before methamphetamine administration [51]. Following methamphetamine dosing, the study subjects were observed for their overall locomotor activity [51]. Immediately after this observation period, the rats’ brains were harvested and brain sections were prepared to highlight nuclei that stained positive for oxytocin and c-Fos, which was used as a marker for overall neural activity [51]. Then, these sections were analyzed for overall nuclei staining positive for c-Fos, oxytocin, or both c-Fos and oxytocin to identify the specific localization of the drug action [51].

Consistent with prior research, study results indicated an overall increase in locomotor activity induced by methamphetamine with attenuation of that activity seen with oxytocin pretreatment [49,51]. However, in this analysis, the rats demonstrated mild sedative responses to oxytocin relative to controls which peaked within the hour following oxytocin administration [51]. This could be a reason why there was a decrease in the self-administration of methamphetamine. In an immunohistological analysis, the results suggested that oxytocin stimulated activity in the hypothalamus and amygdala [51]. Additionally, in the NAc, median preoptic nucleus, and paraventricular thalamic nucleus, methamphetamine and oxytocin appeared to have an additive rather than inhibitory effect on the overall activation [51]. 

### 3.5. Baracz and Parker et al.

Baracz et al. sought to identify changes on the cellular level of the oxytocin receptors (OTR) located within the subthalamic nucleus (STh) and NAc following chronic methamphetamine self-administration and subsequent extinction in rat models [52]. 

During this study, rats were divided into methamphetamine exposure and control groups [52]. While in the study apparatus, the subjects were allowed the freedom to press one of two levers over hours long sessions spanning five days. One lever delivered either intravenous methamphetamine or saline and the other lever did nothing [52]. Following the acquisition of the conditioned response, the rats underwent an extinction period lasting a minimum of ten days [52]. Then, blood was collected from the study subjects to quantify plasma oxytocin and corticosteroid (CORT) levels. At the same time, brain tissue was harvested to identify OTR and corticotrophin-releasing factor (CRF) via immunofluorescence [52]. 

Unsurprisingly, the rats in the methamphetamine exposure group demonstrated rapid acquisition of methamphetamine self-administration relative to the saline controls, along with an overall higher level of locomotor activity, which plateaued on the third day of methamphetamine exposure and remained stable over the following seventeen days [52]. In blood analysis, plasma oxytocin was significantly elevated in the methamphetamine-exposed groups relative to the control groups, while no significant differences in CORT concentration were noted among the experimental arms [52]. The immunohistological analysis demonstrated a significantly higher density of OTRs in the NAc of the control groups relative to the methamphetamine group. At the same time, there was no significant difference in OTR density among the STh specimens [52]. Interestingly, the cumulative methamphetamine intake among study subjects was unrelated to OTR fiber density observed in either the STh or the NAc [52]. In terms of optical analysis, there were significant differences in CRF levels in both the STh and Nac in the treatment groups; however, this was inconsistent with the staining of the brain samples, which demonstrated no significant differences among specimens [52]. 

This study was the first to demonstrate a decrease in OTR density in the NAc along with elevated plasma oxytocin following repeated methamphetamine exposure, both with plasma concentrations remaining elevated and with OTR density slowly returning to baseline following extinction, all of which points toward a modulating effect of oxytocin on methamphetamine abuse [52]. 

## 4. Stauffer et al. and Possible Clinical Efficacy

Stauffer et al. designed a randomized, double-blind control trial to evaluate the effects of oxytocin used with enhanced motivational group therapy on male patients with stimulant use disorder methamphetamine type and a history of sexual interactions with other men (MSM) [55]. All study participants received motivational interviewing group therapy (MIGT), an equal number of subjects in each group being randomly assigned to receive either adjunct oxytocin or a placebo [55]. OT was given intranasally to the subjects. This study involved six sequential group therapy visits, each with a monetary incentive [55]. This study’s primary endpoint was MIGT session attendance with secondary outcomes as follows: group cohesion, anxiety, craving for methamphetamine, use of methamphetamines, and continuous ECG throughout the individual MIGT sessions [55]. 

**Figure 1:**
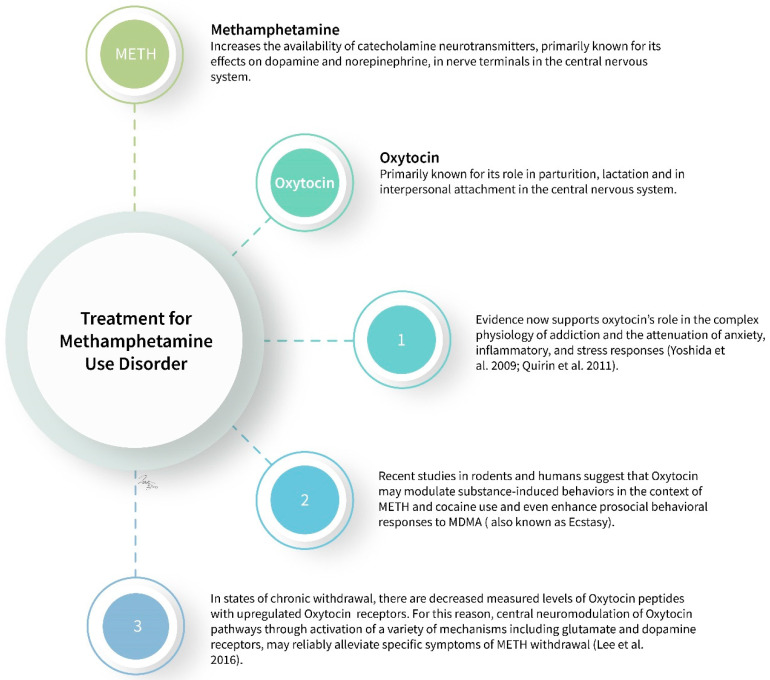
Summary of oxytocin and methamphetamine (METH). Special thanks to our medical illustrator, Rachel Glenn, for the use of this figure in this manuscript.

The forty-eight individuals enrolled in the study were equally and randomly assigned into treatment and placebo groups. Of those, twenty participants in the treatment group completed the study to the final therapy session as compared with nineteen in the placebo group [55]. In all, this study demonstrated that OT led to statistically significant improvements in MIGT session attendance even when controlling for methamphetamine use as measured by urine drug screenings before each session [55]. Additionally, after controlling for methamphetamine use, oxytocin enhancement resulted in small but significant differences in pathophysiological effects, namely lowered heart rates [55]. There were no significant differences noted among the remaining secondary study endpoints, but each did trend against the direction of the effects from the methamphetamine use [55]. This trial represents the first study into the effects of OT on stimulant use disorder methamphetamine type with human subjects; however, due to its limited scope solely on the MSM population, Stauffer et al.’s results have limited generalizability [55]. Figure 1 summarizes the articles discussed in this paper.

## 5. Conclusions

The medical community’s understanding of methamphetamine use has grown significantly in the last two decades, but effective treatment options remain extremely limited. New information regarding the intricacies of methamphetamine’s pathophysiology has led to potential future therapeutics for treating addiction. Because research has shown that oxytocin is extensively involved in many neurochemical pathways, including the central reward system, this neuropeptide has become a notable area of investigation. Contrary to the abovementioned results, one study included in this review suggested that OT may increase drug-seeking behavior [50]. This seemingly happened after the drug was reintroduced by injection in the rodent study. This harkens to the possibility that abstinence from methamphetamine may be needed for efficacy. However, most of the studies regarding oxytocin and methamphetamine were conducted in rodents. More studies on the effects of oxytocin on methamphetamine use should be performed to determine these relationships in human subjects.

Oxytocin has demonstrated the potential to be a useful treatment for methamphetamine use. More human-based research should initiate studies involving the long-term efficacy, side effects, and patient selection. Little is known about the stability of the intranasal administration of oxytocin which was the administration route in the only human study seen thus far. There is a need for more research on other potential oxytocin receptor agonist as well, especially, if oxytocin administration itself does not prove to be stable in humans. Compliance may be another issue that would need to be addressed in future studies. Methamphetamine abuse continues to be a significant cause of distress and dysfunction throughout the world. The effects of oxytocin on methamphetamine use outlined in this review should be a catalyst for further investigation into the efficacy of treatment of methamphetamine use with oxytocin in humans. 

## Data Availability

The data presented in his manuscript can be found in manuscripts indexed in the pubmed database and cited in the references section of this manuscript.
